# Impact of air pollution on stunting among children in Africa

**DOI:** 10.1186/s12940-022-00943-y

**Published:** 2022-12-12

**Authors:** Priyanka N. deSouza, Melanie Hammer, Peter Anthamatten, Patrick L. Kinney, Rockli Kim, S. V. Subramanian, Michelle L. Bell, Kevin M. Mwenda

**Affiliations:** 1 Department of Urban and Regional Planning, University of Colorado Denver, Denver, CO USA; 2 CU Population Center, University of Colorado Boulder, Boulder, CO USA; 3 Department of Energy, Environmental, and Chemical Engineering, Washington University in St. Louis, St. Louis, MO 63130 USA; 4 Department of Physics and Atmospheric Science, Dalhousie University, Halifax, NS Canada; 5 Department of Geography and Environmental Sciences, University of Colorado Denver, Denver, CO USA; 6 Boston University School of Public Health, Boston, MA USA; 7 Division of Health Policy & Management, College of Health Science, Korea University, 145 Anam-ro, Seongbuk-gu, Seoul, 02841 South Korea; 8 Interdisciplinary Program in Precision Public Health, Department of Public Health Sciences, Graduate School of Korea University, Seoul, 02841 South Korea; 9 Harvard Center for Population and Development Studies, Bow Street, Cambridge, MA 02138 USA; 10 Department of Social and Behavioral Sciences, Harvard T.H. Chan School of Public Health, 677 Huntington Avenue, Boston, MA 02115 USA; 11 School of the Environment, Yale University, New Haven, CT USA; 12 Spatial Structures in the Social Sciences, Brown University, Providence, RI USA; 13 Population Studies and Training Center, Brown University, Providence, RI USA

## Abstract

**Background:**

Undernutrition is a global public health crisis, causing nearly half of deaths for children under age 5 years. Little is known regarding the impact of air pollution in-utero and early childhood on health outcomes related to undernutrition. The aim of our study is to evaluate the association of prenatal and early-life exposure to PM_2.5_ and child malnutrition as captured by the height-for-age z-score (HAZ), and stunting in 32 countries in Africa. We also evaluated critical windows of susceptibility during pregnancy to each environmental risk.

**Methods:**

We linked nationally representative anthropometric data from 58 Demographic and Health Surveys (DHS) (*n* = 264,207 children < 5 years of age) with the average in-utero PM_2.5_ concentrations derived from satellite imagery. We then estimated associations between PM_2.5_ and stunting and HAZ after controlling for child, mother and household factors, and trends in time and seasonality.

**Results:**

We observed lower HAZ and increased stunting with higher in-utero PM_2.5_ exposure, with statistically significant associations observed for stunting (OR: 1.016 (95% CI: 1.002, 1.030), for a 10 μg/m^3^ increase). The associations observed were robust to various model specifications. Wald tests revealed that sex, wealth quintile and urban/rural were not significant effect modifiers of these associations. When evaluating associations between trimester-specific PM_2.5_ levels, we observed that associations between PM_2.5_ and stunting was the largest.

**Conclusions:**

This is one of the first studies for the African continent to investigate in-utero and early-life exposure to PM_2.5_ is an important marker of childhood undernutrition. Our results highlight that PM_2.5_ concentrations need to be urgently mitigated to help address undernutrition in children on the continent.

**Supplementary Information:**

The online version contains supplementary material available at 10.1186/s12940-022-00943-y.

## Introduction

Child undernutrition is a major public health crisis. Globally, nearly half of the deaths of children under 5 years of age are caused by poor nutrition. Undernourishment in the first few years of life has been shown to be associated with adverse cognitive health that manifests in lower educational achievement and lower economic productivity later in life, and physical health effects such as lower adult height and higher morbidity and mortality during childhood [2, 3, 7, 17, 24, 25].

The global burden of childhood undernutrition is uneven. According to a 2015 Millennium Development Goals (MDG) report sub-Saharan African accounts for one-third of all undernourished children globally with about 39% stunted, 10% wasted and 25% underweight children under-5 years of age [37]. In light of this, the United Nations Sustainable Development Goal (SDG 2) is to end all forms of malnutrition by 2030 (https://sdgs.un.org/goals/goal2, Last accessed December 16, 2021).

Yet, worsening air pollution can undermine future reductions in undernutrition through direct and indirect effects of health and livelihoods. Population-weighted annual average exposure to PM_2.5_ has increased over the last decade in many African countries [20]. This paper examines associations between exposure to concentrations of fine particulate matter (PM_2.5_), experienced in-utero in 32 African countries and malnutrition in early childhood. Stunting, derived from height-for-age measures, is widely accepted as an indicator of growth and nutrition in children < 5 years of age [8]. We also consider whether factors such as household wealth, urban or rural residence, or sex modify the relationship between PM_2.5_ and malnutrition. Finally, we attempt to identify the critical windows of susceptibility during pregnancy to each air pollution.

### Review of existing evidence that has evaluated associations between PM_2.5_ and post-natal growth

Epidemiologic studies have consistently observed that high concentrations of fine particulate matter (PM_2.5_) has been linked with a wide range of health effects including premature mortality [4, 12], cardiovascular disease [11, 13], respiratory illnesses [14], and cognitive and developmental disorders [5], among others.

Most of the evidence of a link between air pollution and child growth has focused on adverse health outcomes such as early foetal loss [21], and preterm birth, small for gestational age, and low birthweight [9, 15, 30]. Very few studies have evaluated association between ambient air pollution and postnatal growth. One study that used DHS estimates for Bangladesh found that after controlling for other factors that contribute to child anthropometric failure, the relative risks of stunting in the second, third and fourth quartiles of exposure to in-utero PM_2.5_ were 1.074 (95% CI: 1.014, 1.138), 1.150 (95% CI: 1.069, 1.237) and 1.132 (95% CI: 1.031, 1.243), respectively [16]. Another DHS study for India found that after controlling for potential confounders, a 100 μg/m^3^ increase in PM_2.5_ in the month of birth was associated with a 0.05 (95% CI: 0.01–0.09) standard deviation reduction in child height [29]. A final study found that mean in-utero exposure to PM_2.5_ in Indonesia during the 1997 forest fires was associated with a half standard-deviation (0.41) decrease in HAZ at age 17 [31].

### Potential mechanisms underlying the relationship between in-utero and early-life exposure to PM_2.5_ and anthropometric status

Sinharoy et al. [27], found that studies investigating air pollution and intrauterine growth impairment focus on effects at the cellular level. Specifically, these studies hypothesize that exposure to air pollution during pregnancy can cause oxidative stress, which in turn causes inflammation, and potentially poor foetal growth. In addition, they suggest that exposure to particulate matter in-utero modulates DNA methylation, affecting foetal growth. Further, exposure to poor air quality in-utero and in early-life can affect immune ontogeny, which can lead to growth failure in multiple ways. Moreover, children’s lungs are not fully formed until approximately 6 years of age. Exposure to air pollution in young children could affect the formation of the respiratory system, which could in turn affect growth. Finally, the authors posit that air pollution might be responsible for some prenatal vitamin D deficiency, with implications for immune function and bone metabolism. More research is needed to explore these relationships and the critical window of in-utero and early-life exposure to PM_2.5_ that most impacts postnatal growth.

## Methods

### Study population

We drew data from 58 Standard DHSs conducted in 32 countries (Burkina Faso (BF), Benin (BJ), Burundi (BU), Cameroon (CM), Chad (TD), Comoros (KM), Democratic Republic of Congo (CD), Côte d’Ivoire (CI), Eswatini (SZ), Ethiopia (ET), Gabon (GA), Ghana (GH), Guinea (GN), Kenya (KE), Lesotho (LS), Liberia (LB), Madagascar (MD), Malawi (MW), Mali (ML), Mozambique (MZ), Niger (NI), Namibia (NM), Nigeria (NG), Rwanda (RW), Senegal (SN), Sierra Leone (SL), South Africa (ZA), Tanzania (TZ), Togo (TG), Uganda (UG), Zambia (ZM), Zimbabwe (ZW) between 2005 and 2019, where the GPS data were available. We restricted our analysis to DHS surveys for African countries from after 2005, as earlier surveys tend to avoid inconsistencies in measurements, data collection and data reporting.

DHS (https://www.dhsprogram.com/, Last accessed October 18, 2022) are nationally representative household surveys that collect detailed nutrition and health information on children, their parents, and households using a multistage, stratified sampling design. The first stage involves the division of each country into geographic areas. Within these subnational regions, populations are divided into urban and rural areas. These primary sampling units or clusters are selected with probability proportional to the contribution of that cluster’s population to the total population. In the second stage of sampling, all households within the cluster are listed, and an average of 25 houses are randomly selected for an interview with equal-probability systematic sampling.

The eligibility criteria for our analytic sample included survey respondents with:

1) geographic coordinates for each cluster, and 2) complete measures on height-for-age, and 3) same locations for conception and survey interview (when data was collected), 4) estimates for PM_2.5_, and climate data (temperature and precipitation) during the prenatal period and early-life, 5) non-missing information on covariates. Table S1 in Supplementary Information reports the number of children who met these criteria in each of the DHS surveys considered in this study. Our final sample had 264,207 children from 32 countries in Africa (Table 1). Due to the lack of data on important covariates (such as mother’s height, or the outcome variables themselves, we did not include data from other DHS surveys conducted during this time period. For example, the 2003 DHS survey in Ghana did not have information on the anthropometric outcomes considered in this analysis and was therefore, excluded.

**Table 1:** Descriptive Statistics of the DHS variables used as controls in our analysis

DHS Variable definition	Total Sample (%)	Stunted (% of children stunted)	Not Stunted (% of children not stunted)
	246,207 (100%)	99,856 (37.8%)	164,351 (62.2%)
**Urban**	66,253 (25.1%)	18,664 (18.7%)	47,589 (29.0%)
**Male**	132,811 (50.3%)	53,332 (53.4%)	79,479 (48.4%)
**Female**	131,396 (49.7%)	46,524 (46.6%)	84,872 (51.6%)
**High indoor air pollution due to the use of a solid fuel for cooking**	251,836 (95.3%)	97,644 (97.8%)	154,192 (93.8%)
**Wealth quintile 1 (poorest households)**	67,608 (25.6%)	30,006 (30.0%)	37,602 (22.9%)
**Wealth quintile 2**	59,403 (22.5%)	34,360 (34.4%)	25,043 (15.2%)
**Wealth quintile 3**	53,837 (20.4%)	33,062 (33.1%)	20,775 (12.6%)
**Wealth quintile 4**	47,395 (17.9%)	31,651 (31.7%)	15,744 (9.6%)
**Wealth quintile 5 (wealthiest households)**	35,964 (13.6%)	27,676 (27.7%)	8288 (5.0%)
**Maternal Education No formal education**	120,534 (45.6%)	51,638 (51.7%)	68,896 (41.9%)
**Mother Education Primary Education**	91,418 (34.6%)	34,990 (35.0%)	56,428 (34.3%)
**Mother Education Secondary Education**	46,584 (17.6%)	12,433 (12.5%)	34,151 (20.8%)
**Mother Education Advanced**	5671 (2.15%)	795 (0.8%)	4876 (3.0%)
**Maternal height (cm)**	Mean: 158.4 Median: 158.3	Mean: 157.0 Median: 157.0	Mean: 159.3 Median: 159.2
**Maternal BMI (kg/m**^**2**^**)**	Mean: 22.6 Median: 21.8	Mean: 21.9 Median: 21.3	Mean: 23.0 Median: 22.1
**Mother married at < 18 years of age**	137,904 (52.2%)	55,762 (55.8%)	82,142 (50.0%)
**Mother married at ≥ 18 years of age**	126,303 (47.8%)	44,094 (44.2%)	82,209 (50.0%)
**Safe drinking water source if the household had access to piped water into dwelling, yard or plot, public tap or standpipe, tube well or borehole, protected well or spring, rain water, and bottled water**	156,973 (59.4%)	54,843 (54.9%)	102,130 (62.1%)
**No access to Safe drinking water source**	107,234 (40.6%)	45,013 (45.1%)	62,221 (37.9%)
**Household had access to an improved sanitary facility such as access to flush to piped sewer system, septic tank or pit latrine, ventilated improved pit latrine, pit latrine with slab, and composting toilet**	103,183 (39.1%)	34,021 (34.1%)	69,162 (42.1%)
**No access to an improved sanitary facility**	161,024 (60.9%)	65,835 (65.9%)	95,189 (57.9%)
**In-utero PM**_**2.5**_ **(μg/m**^**3**^**)**	Mean: 36.4 Median: 31.1	Mean: 38.9 Median: 32.7	Mean: 34.9 Median: 30.2
**Trimester 1 PM**_**2.5**_ **(**μg**/m**^**3**^**)**	Mean: 36.2 Median: 29.8	Mean: 38.5 Median: 31.0	Mean: 34.8 Median: 29.1
**Trimester 2 PM**_**2.5**_ **(μg/m**^**3**^**)**	Mean: 36.3 Median: 30.3	Mean: 38.8 Median: 31.7	Mean: 34.8 Median: 29.5
**Trimester 3 PM**_**2.5**_ **(μg/m**^**3**^**)**	Mean: 36.6 Median: 30.5	Mean: 39.3 Median: 32.1	Mean: 34.9 Median: 29.6
**Early-life PM**_**2.5**_ **(μg/m**^**3**^**)**	Mean: 35.7 Median: 31.0	Mean: 38.0 Median: 32.6	Mean: 34.3 Median: 30.1
**In-utero Temperature (°C)**	Mean: 24.9 Median: 25.9	Mean: 24.8 Median: 25.8	Mean: 24.9 Median: 26.0
**Trimester 1 Temperature (°C)**	Mean: 24.9 Median: 25.6	Mean: 24.7 Median: 25.4	Mean: 24.9 Median: 25.7
**Trimester 2 Temperature (°C)**	Mean: 24.9 Median: 25.6	Mean: 24.8 Median: 25.4	Mean: 24.9 Median: 25.7
**Trimester 3 Temperature (°C)**	Mean: 24.9 Median: 25.6	Mean: 24.8 Median: 25.5	Mean: 24.9 Median: 25.7
**Early-life Temperature (°C)**	Mean: 24.9 Median: 26.0	Mean: 24.8 Median: 25.9	Mean: 25.0 Median: 26.0
**In-utero Precipitation (mm/month)**	Mean: 95.7 Median: 89.1	Mean: 92.4 Median: 87.0	Mean: 97.7 Median:90.4
**Trimester 1 Precipitation (mm/month)**	Mean: 99.3 Median: 82.1	Mean: 96.8 Median: 80.2	Mean: 101.0 Median: 83.4
**Trimester 2 Precipitation (mm/month)**	Mean: 94.8 Median: 76.3	Mean: 91.7 Median: 73.6	Mean: 96.7 Median: 77.9
**Trimester 3 Precipitation (mm/month)**	Mean: 92.9 Median: 74.3	Mean: 88.7 Median: 69.8	Mean: 95.5 Median: 77.0
**Early-life Precipitation (mm/month)**	Mean: 96.1 Median: 88.5	Mean: 93.2 Median: 87.2	Mean: 97.8 Median: 89.5

Our study followed the Strengthening the Reporting of Observational Studies in Epidemiology (STROBE) reporting guidelines.

#### Stunting

Environmental factors explain more variation in height for children under 5 y of age than ethnic differences. Consequently, child height is a widely accepted indicator of child nutrition. We built our model using the HAZ for children under 5 y of age as the outcome variable, a standardized measure of child heights and a common indicator of stunting. The height-for-age z-score (HAZ) was calculated by comparing the child’s measurements with the median value in the reference population of the National Centre for Health Statistics International Growth Reference. Stunting was defined as a HAZ < -2 standard deviations from the median (WHO Expert Committee on Physical Status: the Use and Interpretation of Anthropometry [34]. Stunting is affected by chronic long-term undernutrition and reflects exposures in-utero, unlike other anthropometric outcomes like underweight and wasting.

### Environmental exposures

#### PM_2.5_ concentrations

**Fig. 1:**
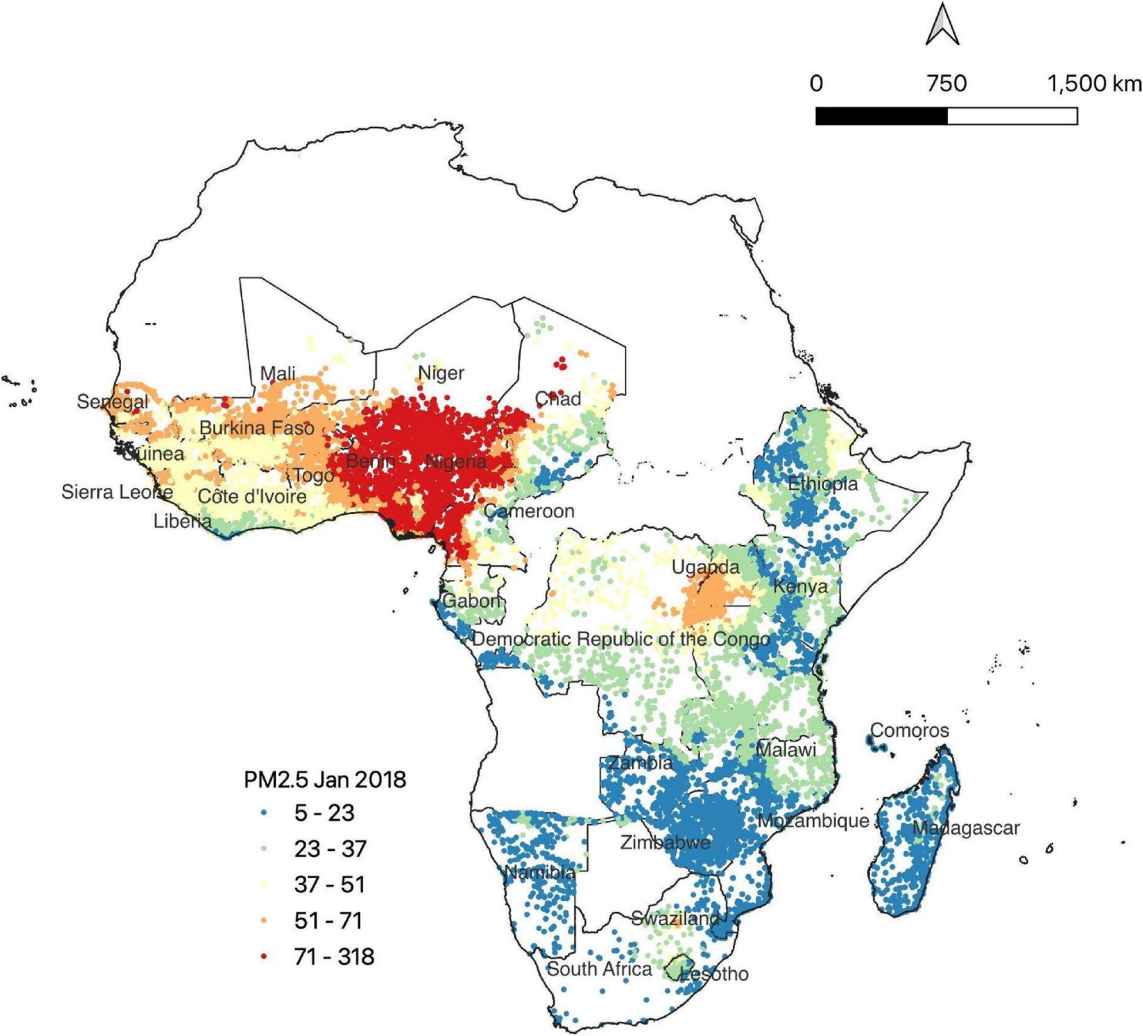
Monthly averaged PM_2.5_ (μg/m^3^) for each cluster in our study for January, 2018 classified into quantiles. The countries we focus on in this study are highlighted

The main exposure variable in this study was long-term ambient PM_2.5_ concentrations. Because most of Africa lacks surface PM_2.5_ monitoring sites at the spatial resolution required for the study, we used satellite-derived monthly PM_2.5_ estimates at a 0.01° × 0.01° (~ 1 km × 1 km at the equator) resolution derived for Africa (Fig. 1) [32]. Satellite aerosol optical depths (AODs) were combined from multiple satellite products: MISR, MODIS Dark Target, MODIS and SeaWiFS Deep Blue, and MODIS MAIAC with simulation-based results based on their relative uncertainties. These AODs were related to near-surface monthly PM_2.5_ concentrations using the ratio of simulated AOD and PM_2.5_ from the GEOS-Chem model. The modeled estimates of PM_2.5_ used in this study agree well with concentrations from global ground-based monitors (R^2^ = 0.81). However, we note that due to the lack of ground-based monitors on the African continent, more validation of the modeled estimates in Africa is needed.

**Fig. 2:**
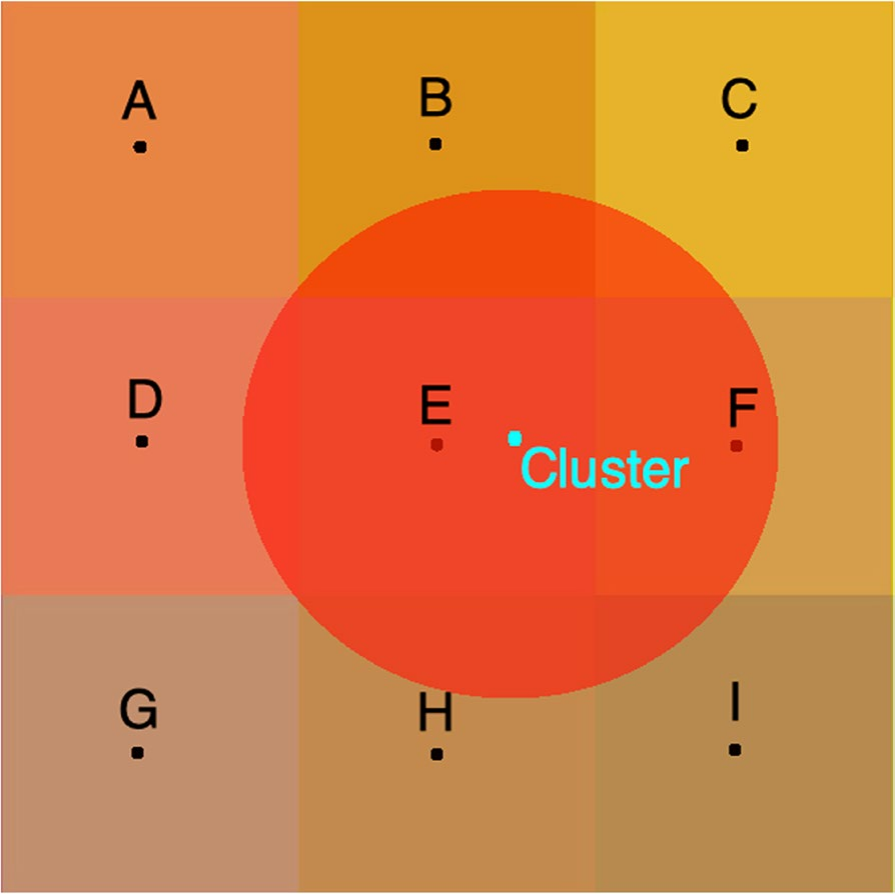
This figure conceptually demonstrates how environmental variables such as PM_2.5_ were joined with DHS sampling clusters. The blue point in the middle represents the sampling cluster. The red circle represents a 2 km urban area buffer around the cluster (note this is 5 km in rural areas). All environmental variables were averaged over the grid cells whose centroids fall within the red circle, which in this figure include cells E and F

Each child was assigned the average ambient PM_2.5_ exposure estimates of members of his/her household cluster, based on GPS coordinates of different household clusters available from the DHS. To maintain the privacy of these respondents, the clusters are randomly displaced by a maximum of 5 km from the true location for rural areas and 2 km in urban areas, with a further 5% of all clusters displaced by 10 km. We extracted the mean PM_2.5_ values from a 2 km buffer around urban clusters and a 5 km buffer around rural clusters (Fig. 2).

We calculated the mean PM_2.5_ exposure corresponding to the 9 months in-utero period (not including month of birth) for the respondent as our main exposure of interest. We also calculated PM_2.5_ exposures for each trimester for each child in the dataset, as well as average PM_2.5_ concentrations experienced in early-life following birth from the month of birth to the month of the DHS interview, which we term as early-life PM_2.5_. The mean age in children in months at the time of the DHS interview was 28.4 (minimum = 0, first quartile = 13, median = 28, third quartile = 43, maximum = 59).

#### Temperature and precipitation

Monthly average temperature and precipitation data during the study period were obtained from the University of East Anglia Climate Research Unit’s Time Series (CRUTS) version 4.05 dataset [19]. CRUTS is a global dataset of monthly weather conditions. The CRUTS data are constructed at a 0.5^o^ resolution and are based on the statistical interpolation of data from over 4000 weather stations, and are considered highly accurate [6, 18, 26, 36]. Mean cluster-level temperature and precipitation were estimated using a spatially-weighted average of the grid cells in the respective buffer around each cluster, in a similar manner to assigning cluster-specific PM_2.5_ exposures. As with PM_2.5_, we also evaluated the mean temperature and precipitation corresponding to the 9 months in-utero, as well as for each trimester, and early-life for every child in our dataset.

### Statistical approach

Characteristics of the 264,207 children in our study were explored using descriptive statistics. We examined correlations between each of the environmental exposures analyzed using Pearson correlations.

We applied a linear model with HAZ as a continuous variable and a logistic model comparing stunted and non-stunted children to evaluate associations with in-utero PM_2.5_. These methodologies have been applied in previous air pollution research [16, 29].

We included the following factors as controls in our model:

1. Child-level characteristics: sex fixed effects, linear and squared terms for the child’s age in months to account for the nonlinear relationship between age and child growth in early childhood [33], birth order, whether the birth was singleton.
2. Maternal characteristics: education level with categories: 1) no education, 2) primary school education, 3) secondary school education, 4) higher); height (cm) and BMI (kg/m^2^), marital status < 18 years of age.
3. Household-level variables: if an improved sanitation facility was present, if a source of safe water was available, if solid fuels were used in the household for cooking, if the household was in an urban or rural environment, and household wealth quintile. The wealth quintile for each household is provided by DHS based on a composite measure of a household’s living standard.
4. Temporal and country fixed effects: birth-year and country-month fixed effects to control for time-varying trends
5. Spatial fixed effects: cluster fixed effects (clusters correspond to villages in rural areas and census enumeration blocks in urban areas) to control for time-invariant cross-region differences (for example higher or lower stunting rates) (Table 1).
6. Meteorological exposures: In-utero and early-life exposure to temperature and precipitation.

This statistical approach was chosen to address sources of confounding. We added the potential confounders in stages to evaluate the robustness of the association between air pollution and HAZ to model specification. We first estimated the model without cluster-level fixed effects. We then added child, mother and household characteristics and meteorological exposures. We report the change in the different outcome parameters for a 10 μg/m^3^ increase in in-utero PM_2.5_ concentrations.

**Fig. 3:**
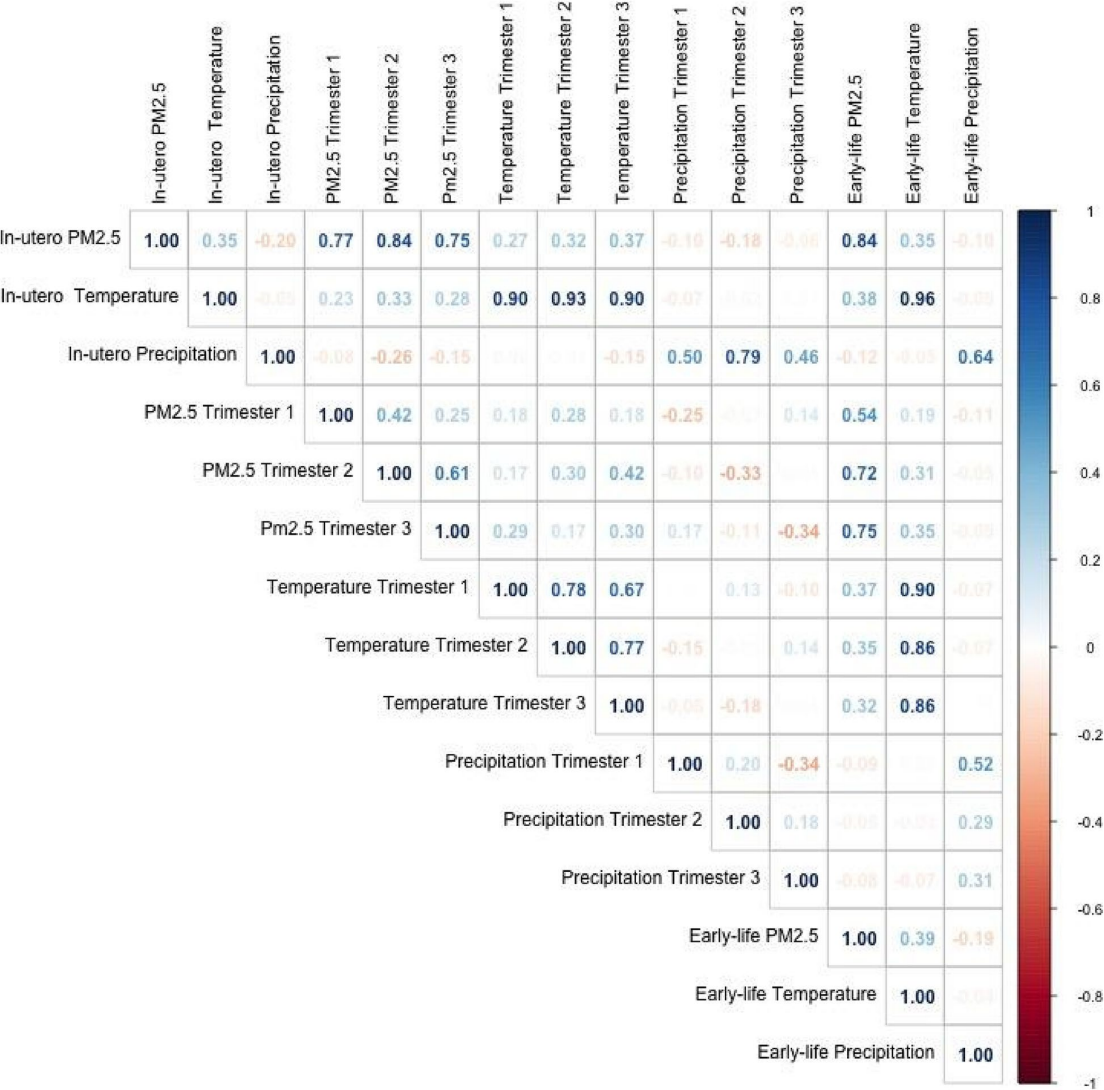
Correlations between in-utero, trimester-specific, and early-life PM_2.5_, temperature and precipitation for the children in our study

The Pearson correlation coefficient between early-life PM_2.5_ concentrations and in-utero PM_2.5_ concentrations are 0.84 (Fig. 3). In sensitivity analyses, we used early-life PM_2.5_ instead of in-utero PM_2.5_ levels as our main exposure of interest. We also applied a model that mutually adjusted for both in-utero PM_2.5_ exposure as well as early-life PM_2.5_ concentrations.

In further sensitivity analyses, we used fixed effects at the household-level instead of at the cluster-level in our fully-adjusted models. In this design, the effects of in-utero PM_2.5_ on HAZ and stunting derive from determining if a child born in a given household during a high period of PM_2.5_ levels is less likely to survive compared to a child born in the same household during a period of low PM_2.5_ concentrations. This design addresses the concern that households with different levels of pollution exposure could be inherently different in unobservable ways.

We tested univariate effect modification of the association by wealth quintile, urban/rural and sex by adding into the model a cross-product term between the modifier and the in-utero PM_2.5_ using Wald tests. We evaluate subgroup-specific associations between in-utero PM_2.5_ and the child growth metrics by including interaction terms between: wealth quintile, urban/rural and sex and in-utero PM_2.5_.

As an additional analysis, we estimated the country-specific relationships between exposure to in-utero PM_2.5_ and HAZ and stunting for each country (except for South Africa where we only have 334 children) in our sample. We then conducted pooled-analyses to combine the country-specific estimates. Estimates are presented for a 10 μg/m^3^ increase in PM_2.5_ to the pooled global estimate and we present these results using a forest plot. In our meta-analysis, the relative contribution (weight) of each country-specific estimate to the pooled estimate is calculated along with the 95% CI from each country-specific analysis. We included random effects in accordance with the DerSimonian and Laird method [10], and we conducted a test of whether the overall pooled estimate is equal to the null as well as a test for heterogeneity, i.e. whether the country-specific estimates are the same. This heterogeneity is quantified using the I-squared measure.

Finally, we developed separate models considering trimester exposures, instead of in-utero PM_2.5_ levels. We controlled for the corresponding trimester temperature and precipitation, and early-life temperature and precipitation in these models. However, because of seasonality bias described in Wilson et al. [35], ,the estimates derived for trimester-specific associations could be biased. Pronounced seasonal variations result in correlations of PM_2.5_ levels between exposure windows (Pearson correlation coefficients range between 0.25 to 0.61) (Fig. 3).

Exposures in other time windows are analogous to confounders, with the possibility that PM_2.5_ in one time period may have an independent association with the outcome, and through its correlations with exposures in other time periods, may confound the observed association. Therefore, we also used the residuals of regressing exposure during each trimester against the other two remaining trimester-specific exposures, as our main exposure of interest. The residuals obtained represent exposure to PM_2.5_ in the given trimester of interest after adjusting for other trimester-specific exposures. This approach avoids covariance among variables representing trimester exposures [1].

We used cluster-adjusted standard errors when reporting results from our models to reflect the assumption that metrics of child growth in the same cluster are not independent. DHS data include sampling weights, to enable generation of representative estimates of the population of the respective country for children under five. Sampling weights are not appropriate for estimating associations [28] as the impact of each environmental exposure on the different administrative units are likely not homogeneous. Sample weights that do not account for such interactions may yield biased associations and so we do not include them in our main analysis.

All statistical tests were 2-tailed, and *p*-values < .05 were considered statistically significant. All analyses were done in R. The main analysis was carried out using the fixest package.

## Results

Information about stunting, PM_2.5_, as well as other covariates were available for 264,207 children across the 58 surveys considered in the study conducted in 32 African countries (Table 1).

Across the entire sample, the average in-utero PM_2.5_ (min = 4.4 μg/m^3^, mean = 36.4 μg/m^3^, median = 21.1 μg/m^3^, max values = 259.4 μg/m^3^), and early-life PM_2.5_ (min = 3.1 μg/m^3^, mean = 35.7 μg/m^3^, median = 31.0 μg/m^3^, max = 233.7 μg/m^3^) varied considerably. Our initial descriptive analysis suggests that stunted children were generally poorer, had mothers of shorter stature, and mainly belonged to rural households, and experienced higher PM_2.5_ concentrations than children who were not stunted (Table 1). The PM_2.5_ exposures considered in this analysis in different windows ang from moderately to highly correlated (Fig. 3).

Table S2 shows results for selected variables from running the linear and logistic models including all variables except for air pollution. Healthier indicators, higher HAZ scores and decreased stunting, were observed among females in comparison to males, for singleton births, for subsequent children after the first birth, for higher educated mothers, for taller mothers, for mothers with a higher BMI, in wealthier households, and in houses with access to improved sanitation.

In Table S3, we present the association and 95% Confidence Interval (CI) between in-utero PM_2.5_ and HAZ, as well as the Odds Ratios (ORs) and 95% CI corresponding to the association between in-utero PM_2.5_ and stunting, adjusting for different sets of covariates. The associations are reported for a 10 μg/m^3^ increase in PM_2.5_. In the simplest models that only adjust for sex and age, we observed significant associations between in-utero PM_2.5_ and the outcomes (Model 1). Further, adjusting for temporal fixed effects (Model 2), strengthened the associations between in-utero PM_2.5_ and stunting and HAZ. However, including DHS cluster fixed effects (Model 3) substantially attenuated the estimated associations. This indicates that seasonality and spatial factors are important confounders of the impact of the different environmental risks on nutrition. By including country-month, year and cluster fixed effects, we adopt a conservative approach to control for residual spatial and temporal confounding in our dataset. The estimates appear to be robust (the change in associations was < 5%) to the inclusion of child-level and maternal (Model 4), household (Model 5) and meteorological covariates (Model 6) (Table S3).

Although we did not observe a significant association between in-utero exposure to PM_2.5_ and HAZ in the fully-adjusted Model 6 displayed in Table 2 (Main Model), the association demonstrated the same general trend of a negative association between in-utero PM_2.5_ and HAZ: -0.003 (95% CI: − 0.012, 0.006). We also observed that in-utero PM_2.5_ was significantly associated with increased stunting OR: 1.017 (95% CI: 1.003, 1.032). The associations observed between in-utero PM_2.5_ and HAZ: -0.005 (95% CI: − 0.014, 0.004) and stunting: OR: 1.017 (95% CI: 1.003, 1.032) remained robust to the inclusion of early-life PM_2.5_ levels in a sensitivity analysis (Table S4).

**Table 2:** Associations (95% Confidence Intervals) between in-utero PM_2.5_ and HAZ, and the Odds Ratios (95% Confidence Intervals) corresponding to associations between in-utero PM_2.5_ exposure and stunting for an increase of 10 μg/m^3^ in PM_2.5_ . Standard errors presented are clustered at the cluster-level

	HAZ	Stunting
**In-utero PM**_**2.5**_	-0.003 (− 0.012, 0.006)	1.016* (1.002, 1.030)
**Controls**	**Sex FE + Age months + Age months**^**2**^ **+ Singleton + country-month fixed effects + year of birth fixed effects + cluster fixed effects + birth order + mother characteristics + household characteristics + temperature and precipitation (in-utero and early-life)**	

*p < 0.05

In supplementary analyses, we observed that when we considered early-life PM_2.5_ exposure instead of in-utero exposure as our main exposure of interest, we observed significant associations with HAZ: -0.033 (95% CI: − 0.059, − 0.008), and indications of a general trend of a positive association with stunting: 1.024 (95% CI: 0.991, 1.059) (Table S5). However, given the strong correlation (0.84, Fig. 3) between in-utero and early-life PM_2.5_ levels, we retain in-utero PM_2.5_ concentrations as our main exposure of interest in this analysis.

In further sensitivity analyses, using household-level fixed effects instead of cluster-level fixed effects (to remove potential omitted variable biases), we observed similar results. The association between in-utero exposure to PM_2.5_ and HAZ was − 0.006 (95% CI: − 0.018, 0.007), and with stunting was: OR: 1.049 (95% CI: 1.009, 1.092) (Table S6). These associations were also robust to the inclusion of early-life PM_2.5_ exposure in models using household-level fixed effects (Table S7). However, only 69,226 households (with a total of 155,317 children) had more than one child. The associations reported in Tables S6 and S7 were for this subset, alone.

Wald tests revealed that child sex, urban/rural and wealth quintile were not significant modifiers of the association between in-utero PM_2.5_ and HAZ or stunting. Subgroup-specific associations are displayed in Table 3. In all subgroups, the confidence intervals overlap widely.

**Table 3:** Subgroup specific associations (95% CI) (for HAZ), and OR (95% CI) (for stunting) between the outcomes of interest and in-utero PM_2.5_ derived by including interaction terms between sex, urban/rural, wealth quintile with in-utero PM_2.5_ in fully-adjusted models (Table 2)

Exposure	HAZ	Stunting
	In-utero PM_**2.5**_	In-utero PM_**2.5**_
**Sex**		
**Male**	-0.005 (− 0.014, 0.004)	1.019 (0.998, 1.028)
**Female**	−0.000 (− 0.010, 0.009)	1.013* (1.005, 1.034)
**Urban/Rural**		
**Urban**	0.006 (−0.013, 0.025)	1.013 (0.999, 1.028)
**Rural**	−0.004 (− 0.013, 0.005)	1.033* (1.002, 1.064)
**Wealth Quintile**		
**1: Poorest**	0.002 (−0.009, 0.013)	1.002 (0.985, 1.018)
**2:**	−0.002 (− 0.014, 0.009)	1.019* (1.002, 1.036)
**3:**	−0.010 (− 0.021, 0.002)	1.023* (1.004, 1.041)
**4:**	−0.008 (− 0.020, 0.003)	1.024* (1.006, 1.044)
**5: Wealthiest**	0.004 (−0.013, 0.020)	1.024* (0.998, 1.051)

*p < 0.05

**Fig. 4:**
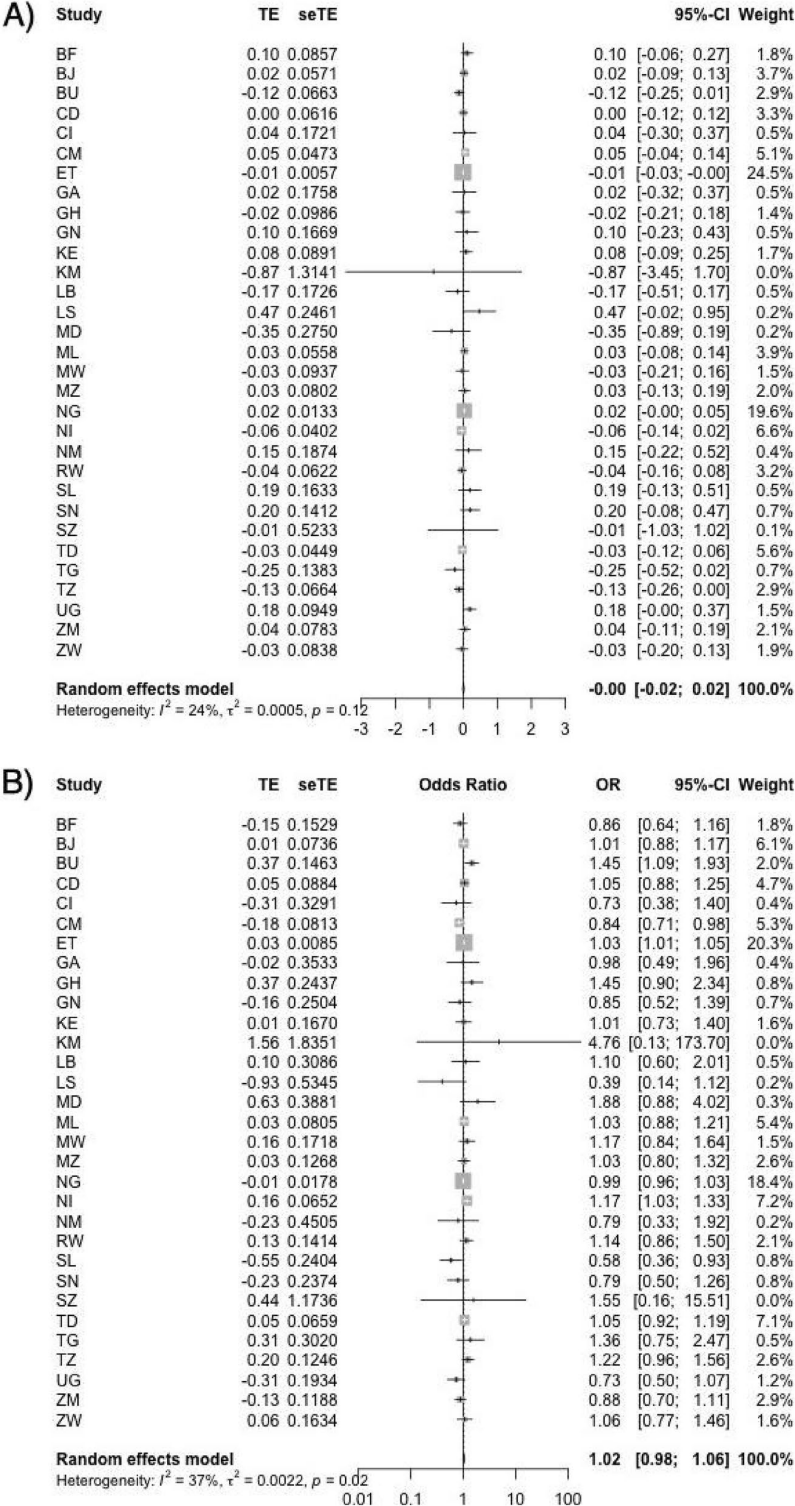
Forest plots from country-specific regression and the pooled analysis for the outcomes HAZ and stunting

We evaluated the relationship between in-utero PM_2.5_ and HAZ and stunting in our study using fully adjusted models for each country in our study (except for South Africa because of the small number of children in the dataset) and then conducted a pooled analysis to compare country-specific results against the summary estimate of the meta-analysis. Forest plots from country-specific regression and the pooled analysis for the outcomes HAZ and stunting are presented in Fig. 4A and B, respectively. The I-squared from these analyses (24 and 37%) suggest that the association for all outcomes within individual countries exhibits moderate variation. Given the large CIs of the associations observed, the results from the pooled analyses suggest that individual country-level studies using DHS data may not have sufficient power to detect a relationship due to the small sample size within each country dataset.

When we evaluated the impact of trimester-specific PM_2.5_ on HAZ and stunting, we observed that exposure to PM_2.5_ in trimester 1, was most strongly associated with increased stunting Model 1, OR: 1.007 (95% CI: 1.002, 1.012). When we accounted for correlations between exposures in different trimesters, by using adjusted trimester exposures as described in Methods, we again fund that PM_2.5_ in trimester 1, was still most strongly associated with stunting OR: 1.006 (95% CI: 1.002, 1.011) (Table 4).

**Table 4:** Associations between the outcomes (HAZ and stunting) and PM_2.5_ experienced during different trimesters from fully-adjusted models

	HAZ	Stunting	Covariates
**Model 1**			
**Trimester 1 PM**_**2.5**_	−0.001 (− 0.004, 0.002)	1.007* (1.002, 1.012)	Child-level + Maternal + Household characteristics + cluster + country-month and year fixed effects + Temperature and Precipitation in trimester 1 + Lifetime Temperature and Precipitation
**Trimester 2 PM**_**2.5**_	0.004 (− 0.004, 0.012)	0.999 (0.988, 1.010)	Child-level + Maternal + Household characteristics + cluster + country-month and year fixed effects + Temperature and Precipitation in trimester 2 + Lifetime Temperature and Precipitation
**Trimester 3 PM**_**2.5**_	−0.004 (− 0.012, 0.004)	1.000 (0.989, 1.012)	Child-level + Maternal + Household characteristics + cluster + country-month and year fixed effects + Temperature and Precipitation in trimester 3 + Lifetime Temperature and Precipitation
**Model 2 (Using residuals of regressing trimester-specific PM**_**2.5**_ **concentrations against concentrations in other trimesters)**			
**Trimester 1 PM**_**2.5**_	−0.002 (− 0.005, 0.001)	1.006* (1.002, 1.011)	Child-level + Maternal + Household characteristics + cluster + country-month and year fixed effects + Temperature and Precipitation in trimester 1 + Lifetime Temperature and Precipitation
**Trimester 2 PM**_**2.5**_	0.005 (− 0.001, 0.011)	0.995 (0.986, 1.003)	Child-level + Maternal + Household characteristics + cluster + country-month and year fixed effects + Temperature and Precipitation in trimester 2 + Lifetime Temperature and Precipitation
**Trimester 3 PM**_**2.5**_	−0.005 (− 0.011, 0.002)	1.000 (0.991, 1.009)	Child-level + Maternal + Household characteristics + cluster + country-month and year fixed effects + Temperature and Precipitation in trimester 3 + Lifetime Temperature and Precipitation

^*^p < 0.05

## Discussion

In this study we evaluated associations between exposure to ambient in-utero PM_2.5_ and stunting and HAZ using data from 58 DHS surveys conducted in 32 African countries between 2005 and 2019.

Using fully adjusted models, we observed a lower HAZ and increased stunting with higher in-utero PM_2.5_ concentrations (Table 2), with statistically significant associations between in-utero PM_2.5_ and stunting OR: 1.016 (95% CI: 1.002, 1.030). The associations were robust to different model specifications (Tables S3, S4), and were also robust when evaluating the impact of PM_2.5_ on children born in the same household (Tables S6, S7).

Child sex, wealth quintile, urban/rural were not significant effect modifiers of the association between in-utero PM_2.5_ and HAZ and stunting. Confidence intervals of the associations derived for subpopulations overlapped widely (Table 3).

We observed moderate heterogeneity in the association between PM_2.5_ and stunting in our country-specific analyses (Fig. 4), likely due to different baseline policies that impact stunting such as access to nutrition (food prices, food availability), health care systems, etc., as well as differences in activity patterns occupational exposures, other population characteristics, built environment, and particulate matter chemical composition. Other studies have demonstrated differences in the health associations for particulate matter in different locations [22, 23]. However, due to the limited number of observations within each country, we do not have the statistical power to detect robust relationships between PM_2.5_ and stunting or HAZ.

When evaluating trimester-specific associations, we also observed the highest associations between PM_2.5_ in trimester 1 (but not trimesters 2 and 3) and stunting, even after adjusting for exposures in other time windows and other covariates (Table 4). This suggests that PM_2.5_ experienced in early pregnancy likely has the greatest impact on stunting. More work is needed to evaluate critical windows of exposure to PM_2.5_ for HAZ and stunting.

Our study has several limitations. First, the dataset does not include a measure of gestational age, and therefore, assume that each child in our sample is carried to term, which is reflected in our calculation of in-utero exposures. Second, our results may suffer from residual confounding from omitted variables that are correlated both with the environmental exposures and the outcomes considered in this study. Third, the exposure of interest in our analysis is PM_2.5_, which is based on modelled data rather than monitoring data. Although this dataset has been validated using the global distribution of ground-based monitors (R^2^ = 0.81), there is a lack of surface-monitors in Africa and more research is required to evaluate the modeled estimates in the countries considered in this study. More work is also needed to examine the sensitivity of the calculated health impact of the different environmental exposures to the exposure product used (for example in deSouza et al. [12],). Fourth, our sample pools together data on all births in the 5 years preceding the survey date for women of reproductive age (15–49 years) who lived in the sampled households. Since these data on births are reported by mothers, however, we may be missing data on children in the households whose mothers have died or who were not present at the time of the survey. Sixth, research has shown that anthropometry is a complex indicator that captures genetic, environmental, behavioral factors, as well as exposure to disease. The health outcome: anthropometric failure must be complemented by other diet and food based measures in future work to measure the impact of PM_2.5_ on undernutrition. Seventh, the study cannot disentangle the impacts of different particulate matter chemical composition and sources, which are likely to vary across the study area due to different sources, meteorology, etc. Eighth, the population characteristics considered are not the same across all the study locations, and the impact of PM_2.5_ is likely different in different locations. Finally, while this is one of the first (perhaps the first) to investigate this topic for Africa, even so large parts of the continent are not included and more work is needed on these other regions. Nevertheless, our study provides important insights into the relationship between air pollution and stunting, and demonstrates the importance of reducing PM_2.5_ concentrations in African countries to protect children.

## Supplementary Information

**Additional file 1: Table S1.** Study sample with filtered observations by country. **Table S2.** Associations (95% Confidence Intervals) between in-utero PM_2.5_ and HAZ, and the Odds Ratios (95% Confidence Intervals) corresponding to associations between in-utero PM_2.5_ exposure and stunting for an increase of 10 μg/m^3^ in PM_2.5_. Standard errors presented are clustered at the cluster-level. **Table S3.** Associations (95% Confidence Intervals) between in-utero PM_2.5_ and HAZ, and the Odds Ratios (95% Confidence Intervals) corresponding to associations between in-utero PM_2.5_ and stunting for an increase of 10 μg/m3 in PM_2.5_. Standard errors presented are clustered at the cluster-level. Note that sample sizes vary across Models because some fixed effects categories lack within-category variation in the independent variable (resulting in that category being dropped). The main model is highlighted. **Table S4.** Associations (95% Confidence Intervals) between in-utero and early-life PM_2.5_ and HAZ, and the Odds Ratios (95% Confidence Intervals) corresponding to associations between in-utero and early-life PM_2.5_ and stunting for an increase of 10 μg/m3 in PM_2.5_. Standard errors presented are clustered at the cluster-level. Note that sample sizes vary across Models because some fixed effects categories lack within-category variation in the independent variable (resulting in that category being dropped). . The main model is highlighted. **Table S5.** Associations (95% Confidence Intervals) between early-life PM_2.5_ and HAZ, and the Odds Ratios (95% Confidence Intervals) corresponding to associations between early-life PM_2.5_ and stunting for an increase of 10 μg/m3 in PM_2.5_. Standard errors presented are clustered at the cluster-level. Note that sample sizes vary across Models because some fixed effects categories lack within-category variation in the independent variable (resulting in that category being dropped). . The main model is highlighted. **Table S6.** Associations (95% Confidence Intervals) between in-utero PM_2.5_ and HAZ, and the Odds Ratios (95% Confidence Intervals) corresponding to associations between in-utero PM_2.5_ and stunting for an increase of 10 μg/m3 in PM_2.5_ derived from fully-adjusted models (Model 6 in Table S3), using household-fixed effects instead of cluster-specific fixed effects. **Table S7.** Associations (95% Confidence Intervals) between in-utero and early-life PM_2.5_ and HAZ, and stunting for an increase of 10 μg/m3 in PM_2.5_ derived from fully-adjusted models (Model 6 in Table S4), using household-fixed effects instead of cluster-specific fixed effects.
