# Supplementary material for: Impact of air pollution on stunting among children in Africa
Source: Environ Health. 2022 Dec 12;21:128. doi: 10.1186/s12940-022-00943-y (PMC9743768; doi:10.1186/s12940-022-00943-y)
Supplement: Supplementary file 1 — Additional file 1: Table S1. Study sample with filtered observations by country. Table S2. Associations (95% Confidence Intervals) between in-utero PM2.5 and HAZ, and the Odds Ratios (95% Confidence Intervals) corresponding to associations between in-utero PM2.5 exposure and stunting for an increase of 10 μg/m3 in PM2.5. Standard errors presented are clustered at the cluster-level. Table S3. Associations (95% Confidence Intervals) between in-utero PM2.5 and HAZ, and the Odds Ratios (95% Confidence Intervals) corresponding to associations between in-utero PM2.5 and stunting for an increase of 10 μg/m3 in PM2.5. Standard errors presented are clustered at the cluster-level. Note that sample sizes vary across Models because some fixed effects categories lack within-category variation in the independent variable (resulting in that category being dropped). The main model is highlighted. Table S4. Associations (95% Confidence Intervals) between in-utero and early-life PM2.5 and HAZ, and the Odds Ratios (95% Confidence Intervals) corresponding to associations between in-utero and early-life PM2.5 and stunting for an increase of 10 μg/m3 in PM2.5. Standard errors presented are clustered at the cluster-level. Note that sample sizes vary across Models because some fixed effects categories lack within-category variation in the independent variable (resulting in that category being dropped). . The main model is highlighted. Table S5. Associations (95% Confidence Intervals) between early-life PM2.5 and HAZ, and the Odds Ratios (95% Confidence Intervals) corresponding to associations between early-life PM2.5 and stunting for an increase of 10 μg/m3 in PM2.5. Standard errors presented are clustered at the cluster-level. Note that sample sizes vary across Models because some fixed effects categories lack within-category variation in the independent variable (resulting in that category being dropped). . The main model is highlighted. Table S6. Associations (95% Confidence Intervals) [file 12940_2022_943_MOESM1_ESM.docx]

Supplementary Information:

Impact of Air Pollution on Stunting Among Children in Africa

Priyanka N deSouza^1,2*^, Melanie Hammer^3,4^, Peter Anthamatten^5^ , Patrick L Kinney^6^ , Rockli Kim^7,8,9^ , S.V Subramanian^9,10^, Michelle L Bell^11^, Kevin M Mwenda^12,13^

^1^: Department of Urban and Regional Planning, University of Colorado Denver, Denver, CO, USA

^2^: CU Population Center, University of Colorado Boulder, Boulder CO, USA

^3^:Department of Energy, Environmental, and Chemical Engineering, Washington University in St. Louis, St. Louis, MO 63130, USA.

^4^: Department of Physics and Atmospheric Science, Dalhousie University, Halifax, NS, Canada.

^5^: Department of Geography and Environmental Sciences, University of Colorado Denver, Denver, CO, USA

^6^: Boston University School of Public Health, Boston, MA, USA

^7^: Division of Health Policy & Management, College of Health Science, Korea University, 145 Anam-ro, Seongbuk-gu, Seoul, 02841, South Korea

^8^: Interdisciplinary Program in Precision Public Health, Department of Public Health Sciences, Graduate School of Korea University, Seoul, 02841, South Korea

^9^: Harvard Center for Population and Development Studies, Bow Street, Cambridge, MA, 02138, USA

^10^: Department of Social and Behavioral Sciences, Harvard T.H. Chan School of Public Health, 677 Huntington Avenue, Boston, MA, 02115, USA

^11^:School of the Environment, Yale University, New Haven, CT, USA

^12^: Spatial Structures in the Social Sciences, Brown University, Providence, RI, USA

^13^: Population Studies and Training Center, Brown University, Providence, RI USA

*: Corresponding author

priyanka.desouza@ucdenver.edu

***Table S1****: Study sample with filtered observations by country*

| **Country** | **Country Code** | **DHS Year** | **Total number of children** | **Number of children alive** | **Number of children born in same cluster** | **Valid HAZ** | **Valid GPS coordinates** | **Valid exposure data** | **Valid covariates** |
| --- | --- | --- | --- | --- | --- | --- | --- | --- | --- |
| Burkino Faso | BF | 2010 | 15044 | 13716 | 6532 | 6152 | 6152 | 6152 | 5994 |
| Benin | BJ | 2017 - 2018 | 13589 | 12651 | 11765 | 10939 | 10709 | 10709 | 5431 |
| Benin | BJ | 2011-2012 | 13407 | 12679 | 12679 | 7636 | 7593 | 7593 | 7328 |
| Burundi | BU | 2016-2017 | 13192 | 12472 | 11611 | 5641 | 5613 | 5613 | 5433 |
| Burundi | BU | 2010-2011 | 7742 | 7231 | 7231 | 3450 | 3450 | 3450 | 3330 |
| Cameroon | CM | 2018 | 9733 | 9085 | 7292 | 3632 | 3632 | 3632 | 3134 |
| Cameroon | CM | 2011 | 11732 | 10734 | 10734 | 5033 | 5031 | 5031 | 4443 |
| Chad | TD | 2014-2015 | 18623 | 16901 | 16901 | 9826 | 9826 | 9826 | 9572 |
| Comoros | KM | 2012 | 3149 | 3022 | 3022 | 2387 | 2278 | 2278 | 1920 |
| Congo Democratic Republic | CD | 2013-2014 | 18716 | 17228 | 17228 | 8059 | 7415 | 7415 | 6946 |
| Congo Democratic Republic | CD | 2007 | 8992 | 7987 | 7256 | 3003 | 2937 | 2937 | 2779 |
| Cote d'Ivoire | CI | 2011-2012 | 7776 | 7093 | 7093 | 3200 | 3108 | 3108 | 2646 |
| Eswatini | SZ | 2006-2007 | 2812 | 2537 | 1924 | 1670 | 1636 | 1636 | 1006 |
| Ethiopia | ET | 2016 | 10641 | 10006 | 9597 | 8537 | 8213 | 8213 | 7993 |
| Ethiopia | ET | 2011 | 11654 | 10808 | 10808 | 9611 | 9297 | 9252 | 8868 |
| Gabon | GA | 2012 | 6067 | 5747 | 5747 | 3336 | 3312 | 3312 | 2619 |
| Ghana | GH | 2014 | 5884 | 5595 | 5595 | 2720 | 2686 | 2686 | 2456 |
| Ghana | GH | 2008 | 2992 | 2794 | 2348 | 2021 | 1990 | 1990 | 1884 |
| Guinea | GN | 2018 | 7951 | 7273 | 6771 | 3247 | 3247 | 3247 | 3111 |
| Guinea | GN | 2012 | 7039 | 6424 | 6424 | 3085 | 3085 | 3085 | 2900 |
| Kenya | KE | 2014 | 20964 | 20093 | 18457 | 17270 | 17180 | 17180 | 6878 |
| Kenya | KE | 2008-2009 | 6079 | 5706 | 4711 | 4256 | 4240 | 4240 | 3836 |
| Lesotho | LS | 2014 | 3138 | 2915 | 2915 | 1312 | 1312 | 1312 | 1042 |
| Lesotho | LS | 2009-2010 | 3999 | 3606 | 2735 | 1289 | 1275 | 1275 | 1042 |
| Liberia | LB | 2019 | 5704 | 5245 | 4814 | 2277 | 2252 | 2252 | 1799 |
| Liberia | LB | 2013 | 7606 | 7058 | 7058 | 3171 | 3171 | 3171 | 2717 |
| Madagascar | MD | 2008-2009 | 12448 | 11750 | 10813 | 4524 | 4447 | 4447 | 4246 |
| Malawi | MW | 2015-2016 | 17286 | 16462 | 14337 | 4570 | 4570 | 4570 | 4391 |
| Malawi | MW | 2010 | 19967 | 18360 | 16152 | 4035 | 3949 | 3949 | 3851 |
| Mali | ML | 2018 | 9940 | 9275 | 8908 | 8013 | 7759 | 7759 | 3812 |
| Mali | ML | 2012-2013 | 10326 | 9582 | 9582 | 4306 | 4306 | 4306 | 4206 |
| Mali | ML | 2006 | 14238 | 12437 | 11762 | 10261 | 10234 | 10234 | 10086 |
| Mozambique | MZ | 2011 | 11102 | 10291 | 10291 | 9313 | 9313 | 9304 | 8693 |
| Niger | NI | 2012 | 12558 | 11602 | 11602 | 4771 | 4771 | 4771 | 4672 |
| Namibia | NM | 2013 | 5046 | 4818 | 4818 | 1558 | 1558 | 1558 | 876 |
| Namibia | NM | 2006-2007 | 5168 | 4858 | 3692 | 2965 | 2908 | 2908 | 1739 |
| Nigeria | NG | 2018 | 33924 | 30713 | 28380 | 10389 | 10337 | 10337 | 9018 |
| Nigeria | NG | 2013 | 31482 | 28596 | 28596 | 24505 | 24311 | 24311 | 19513 |
| Nigeria | NG | 2008 | 28647 | 25446 | 22558 | 16929 | 16915 | 16915 | 14353 |
| Rwanda | RW | 2019 | 8092 | 7796 | 6329 | 3143 | 3143 | 3143 | 2835 |
| Rwanda | RW | 2014-2015 | 7856 | 7558 | 7558 | 3538 | 3538 | 3538 | 3202 |
| Rwanda | RW | 2010-2011 | 9002 | 8484 | 8484 | 4075 | 4075 | 4075 | 3780 |
| Senegal | SN | 2010-2011 | 12326 | 11633 | 11633 | 3548 | 3476 | 3476 | 3236 |
| Sierra Leone | SL | 2019 | 9899 | 9063 | 8195 | 3773 | 3629 | 3629 | 3211 |
| Sierra Leone | SL | 2013 | 11938 | 10618 | 10618 | 4069 | 4069 | 4069 | 3705 |
| Sierra Leone | SL | 2008 | 5631 | 5043 | 4420 | 1791 | 1780 | 1780 | 1604 |
| South Africa | ZA | 2016 | 3548 | 3413 | 2859 | 973 | 973 | 972 | 334 |
| Tanzania | TZ | 2015-2016 | 10233 | 9713 | 8364 | 7897 | 7897 | 7897 | 7309 |
| Tanzania | TZ | 2009-2010 | 8023 | 7526 | 7526 | 6792 | 6561 | 6537 | 5977 |
| Togo | TG | 2013-2014 | 6979 | 6535 | 6535 | 3185 | 3185 | 3185 | 3013 |
| Uganda | UG | 2016 | 15522 | 14710 | 11398 | 3573 | 3518 | 3518 | 3349 |
| Uganda | UG | 2011 | 7878 | 7355 | 7355 | 2070 | 2042 | 2042 | 1934 |
| Uganda | UG | 2006 | 8369 | 7593 | 5829 | 1899 | 1741 | 1741 | 1685 |
| Zambia | ZM | 2013-2014 | 13457 | 12714 | 9850 | 8946 | 8914 | 8914 | 8052 |
| Zambia | ZM | 2007 | 6401 | 5844 | 4407 | 3919 | 3919 | 3919 | 3634 |
| Zimbabwe | ZW | 2015 | 6132 | 5807 | 4581 | 4060 | 4060 | 4060 | 3669 |
| Zimbabwe | ZW | 2010-2011 | 5563 | 5203 | 5203 | 4299 | 4156 | 4156 | 3785 |
| Zimbabwe | ZW | 2005-2006 | 5246 | 4875 | 4457 | 3607 | 3581 | 3581 | 3330 |
| Total |  |  | 618452 | 572279 | 526340 | 314056 | 310275 | 310196 | 264207 |

***Table S2****: Associations (95% Confidence Intervals) between in-utero PM_2.5_ and HAZ, and the Odds Ratios (95% Confidence Intervals) corresponding to associations between in-utero PM_2.5_ exposure and stunting for an increase of 10 μg/m^3^ in PM_2.5_ . Standard errors presented are clustered at the cluster-level.*

|  | **HAZ** | **Stunting** |
| --- | --- | --- |
|  | |  |
| **Sex** |  |  |
| **Female: Reference** |  |  |
| **Male** | -0.177*  (-0.190, -0.165) | 1.331*  (1.304, 1.359) |
| **Age (months)** | -0.120*  (-0.149, -0.091) | 1.149*  (1.100, 1.201) |
| **Age^2^ (months)** | 0.001*  (0.001, 0.001) | 0.998*  (0.998, 0.998) |
| **Singleton Birth** |  |  |
| **No: Reference** |  |  |
| **Yes** | 0.671*  (0.624, 0.719) | 0.367*  (0.340, 0.396) |
| **Birth order** | -0.006*  (-0.009, -0.003) | 1.013*  (1.008, 1.018) |
| **Mother Education Level** |  |  |
| **No formal education: Reference** |  |  |
| **Primary** | -0.001  (-0.021, 0.019) | 0.999  (0.968, 1.031) |
| **Secondary** | 0.069*  (0.043, 0.095) | 0.850*  (0.814, 0.889) |
| **Higher** | 0.235*  (0.179, 0.292) | 0.622*  (0.549, 0.705) |
| **Mother Married < 18 y of age** |  |  |
| **No: Reference** |  |  |
| **Yes** | 0.001  (-0.014, 0.016) | 0.996  (0.971, 1.021) |
| **Mother height** | 0.041*  (0.039, 0.042) | 0.940*  (0.938, 0.942) |
| **Mother BMI** | 0.034*  (0.032, 0.036) | 0.949*  (0.945, 0.952) |
| **Wealth Quintile** |  |  |
| **1: Reference** |  |  |
| **2** | 0.065*  (0.042, 0.088) | 0.894*  (0.864, 0.925) |
| **3** | 0.120*  (0.095, 0.146) | 0.811*  (0.780, 0.844) |
| **4** | 0.191*  (0.162, 0.220) | 0.707*  (0.675, 0.742) |
| **5** | 0.325*  (0.284, 0.365) | 0.563*  (0.525, 0.604) |
| **Access to Safe Drinking Water** |  |  |
| **No: Reference** |  |  |
| **Yes** | -0.012  (-0.034, 0.010) | 1.006  (0.973, 1.040) |
| **Access to Improved Sanitation** |  |  |
| **No: Reference** |  |  |
| **Yes** | 0.041*  (0.020, 0.063) | 0.949*  (0.916, 0.982) |
| **Urban/Rural** |  |  |
| **Rural: Reference** |  |  |
| **Urban** | -3.248  (-18500, 18500) | 1.231  (0, ∞) |
| **In-utero Temperature** | 0.006  (-0.018, 0.031) | 0.952*  (0.916, 0.989) |
| **In-utero Precipitation** | 4.1 x 10^-4^  (-0.83 x 10^-4^ , 9.0 x 10^-4^) | 0.999  (0.998, 1.000) |
| **Early-life Temperature** | 0.034*  (0.016, 0.052) | 0.974  (0.945, 1.003) |
| **Early-life Precipitation** | 0.36 x 10^-4^  (-3.51 x 10^-4^, 4.23 x 10^-4^) | 1.000  (0.999, 1.000) |
| **Additional controls** | **country month fixed effects + year of birth fixed effects + cluster fixed effects** | |

*(*: p< 0.05)*

***Table S3****: Associations (95% Confidence Intervals) between in-utero PM_2.5_ and HAZ, and the Odds Ratios (95% Confidence Intervals) corresponding to associations between in-utero PM_2.5_ and stunting for an increase of 10 μg/m^3^ in PM_2.5_ . Standard errors presented are clustered at the cluster-level. Note that sample sizes vary across Models because some fixed effects categories lack within-category variation in the independent variable (resulting in that category being dropped). The main model is highlighted.*

|  | **Model 1** | **Model 2** | **Model 3** | **Model 4** | **Model 5** | **Main Model**  **Model 6** |
| --- | --- | --- | --- | --- | --- | --- |
| **Outcome: HAZ (coefficient)** | | | | | | |
| **In-utero PM_2.5_** | -0.067*  (-0.070, -0.064) | -0.120*  (-0.125, -0.114) | -0.004  (-0.013, 0.005) | -0.003  (-0.012, 0.006) | -0.003  (-0.12, 0.005) | -0.003  (-0.012, 0.006) |
| **Outcome: Stunting (OR)** | | | | | | |
| **In-utero PM_2.5_** | 1.020*  (1.019, 1.021) | 1.157*  (1.150, 1.165) | 1.017*  (1.004, 1.031) | 1.017*  (1.003, 1.031) | 1.018*  (1.004, 1.032) | 1.016*  (1.002, 1.030) |
| **Sex FE + Age months + Age months^2^ + Singleton** | yes | yes | yes | yes | yes | yes |
| **country-month FEs** |  | yes | yes | yes | yes | yes |
| **year of birth FEs** |  | yes | yes | yes | yes | yes |
| **cluster fixed effect** |  |  | yes | yes | yes | yes |
| **birth order + mother characteristics** |  |  |  | yes | yes | yes |
| **household characteristics** |  |  |  |  | yes | yes |
| **Temperature + Precipitation (In-utero and Early-life)** |  |  |  |  |  | yes |

***Table S4****: Associations (95% Confidence Intervals) between in-utero and early-life PM_2.5_ and HAZ, and the Odds Ratios (95% Confidence Intervals) corresponding to associations between in-utero and early-life PM_2.5_ and stunting for an increase of 10 μg/m^3^ in PM_2.5_ . Standard errors presented are clustered at the cluster-level. Note that sample sizes vary across Models because some fixed effects categories lack within-category variation in the independent variable (resulting in that category being dropped). . The main model is highlighted.*

|  | **Model 1** | **Model 2** | **Model 3** | **Model 4** | **Model 5** | **Main Model**  **Model 6** |
| --- | --- | --- | --- | --- | --- | --- |
| **Outcome: HAZ (coefficient)** | | | | | | |
| **In-utero PM_2.5_** | -0.040*  (-0.046, -0.035) | -0.048*  (-0.056, -0.040) | -0.006  (-0.015, 0.003) | -0.005  (-0.014, 0.004) | -0.005  (-0.014, 0.003) | -0.005  (-0.014, 0.004) |
| **Early-Life PM_2.5_** | -0.036*  (-0.042, -0.029) | -0.107*  (-0.117, -0.098) | -0.027*  (-0.050, -0.004) | -0.026*  (-0.049, -0.003) | -0.026*  (-0.048, -0.003) | -0.035*  (-0.060, -0.009) |
| **Outcome: Stunting (OR)** | | | | | | |
| **In-utero PM_2.5_** | 1.011*  (1.009, 1.013) | 1.062*  (1.052, 1.073) | 1.019*  (1.005, 1.033) | 1.019*  (1.005, 1.033) | 1.020*  (1.006, 1.034) | 1.017*  (1.003, 1.032) |
| **Early-Life PM_2.5_** | 1.012*  (1.011, 1.014) | 1.135*  (1.122, 1.148) | 1.024  (0.994, 1.054) | 1.024  (0.994, 1.055) | 1.024  (0.994, 1.055) | 1.029  (0.995, 1.065) |
| **Sex FE + Age months + Age months^2^ + Singleton** | yes | yes | yes | yes | yes | yes |
| **country-month FEs** |  | yes | yes | yes | yes | yes |
| **year of birth FEs** |  | yes | yes | yes | yes | yes |
| **cluster fixed effect** |  |  | yes | yes | yes | yes |
| **birth order + mother characteristics** |  |  |  | yes | yes | yes |
| **household characteristics** |  |  |  |  | yes | yes |
| **Temperature + Precipitation (In-utero and Early-life)** |  |  |  |  |  | yes |

***Table S5****: Associations (95% Confidence Intervals) between early-life PM_2.5_ and HAZ, and the Odds Ratios (95% Confidence Intervals) corresponding to associations between early-life PM_2.5_ and stunting for an increase of 10 μg/m^3^ in PM_2.5_ . Standard errors presented are clustered at the cluster-level. Note that sample sizes vary across Models because some fixed effects categories lack within-category variation in the independent variable (resulting in that category being dropped). . The main model is highlighted.*

|  | **Model 1** | **Model 2** | **Model 3** | **Model 4** | **Model 5** | **Main Model**  **Model 6** |
| --- | --- | --- | --- | --- | --- | --- |
| **Outcome: HAZ (coefficient)** | | | | | | |
| **Early-life PM_2.5_** | -0.073*  (-0.077, -0.070) | -0.149*  (-0.156, -0.143) | -0.025*  (-0.048, -0.002) | -0.025*  (-0.048, -0.002) | -0.024*  (-0.047, -0.002) | -0.033*  (-0.059, -0.008) |
| **Outcome: Stunting (OR)** | | | | | | |
| **Early-life PM_2.5_** | 1.023*  (1.022, 1.024) | 1.197*  (1.189, 1.206) | 1.018  (0.989, 1.048) | 1.019  (0.990, 1.050) | 1.019  (0.989, 1.050) | 1.024  (0.991, 1.059) |
| **Sex FE + Age months + Age months^2^ + Singleton** | yes | yes | yes | yes | yes | yes |
| **country-month FEs** |  | yes | yes | yes | yes | yes |
| **year of birth FEs** |  | yes | yes | yes | yes | yes |
| **cluster fixed effect** |  |  | yes | yes | yes | yes |
| **birth order + mother characteristics** |  |  |  | yes | yes | yes |
| **household characteristics** |  |  |  |  | yes | yes |
| **Temperature + Precipitation (In-utero and Early-life)** |  |  |  |  |  | yes |

***Table S6***: *: Associations (95% Confidence Intervals) between in-utero PM_2.5_ and HAZ, and the Odds Ratios (95% Confidence Intervals) corresponding to associations between in-utero PM_2.5_ and stunting for an increase of 10 μg/m^3^ in PM_2.5_  derived from fully-adjusted models (Model 6 in Table S3), using household-fixed effects instead of cluster-specific fixed effects.*

|  | **Outcome: HAZ** |
| --- | --- |
| **In-utero PM_2.5_** | -0.006  (-0.018, 0.007) |
|  | **Outcome: Stunting** |
| **In-utero PM_2.5_** | 1.049*  (1.009, 1.092) |

*(*: p< 0.05)*

***Table S7***: *Associations (95% Confidence Intervals) between in-utero and early-life PM_2.5_ and HAZ, and stunting for an increase of 10 μg/m^3^ in PM_2.5_  derived from fully-adjusted models (Model 6 in Table S4), using household-fixed effects instead of cluster-specific fixed effects.*

|  | **Outcome: HAZ** |
| --- | --- |
| **In-utero PM_2.5_** | -0.005  (-0.018, 0.007) |
| **Early-life PM_2.5_** | -0.051*  (-0.078, -0.024) |
|  | **Outcome: Stunting** |
| **In-utero PM_2.5_** | 1.048*  (1.007, 1.090) |
| **Early-life PM_2.5_** | 1.084*  (1.003, 1.172) |

*(*: p< 0.05)*
